# Convolutional Sparse Support Estimator-Based COVID-19 Recognition From X-Ray Images

**DOI:** 10.1109/TNNLS.2021.3070467

**Published:** 2021-04-19

**Authors:** Mehmet Yamaç, Mete Ahishali, Aysen Degerli, Serkan Kiranyaz, Muhammad E. H. Chowdhury, Moncef Gabbouj

**Affiliations:** Faculty of Information Technology and Communication SciencesTampere University 7840 33720 Tampere Finland; Department of Electrical EngineeringQatar University 61780 Doha 2713 Qatar

**Keywords:** Coronavirus disease (COVID-19) recognition, representation-based classification, severe acute respiratory syndrome coronavirus 2 (SARS-CoV-2)~virus, transfer learning

## Abstract

Coronavirus disease (COVID-19) has been the main agenda of the whole world ever since it came into sight. X-ray imaging is a common and easily accessible tool that has great potential for COVID-19 diagnosis and prognosis. Deep learning techniques can generally provide state-of-the-art performance in many classification tasks when trained properly over large data sets. However, data scarcity can be a crucial obstacle when using them for COVID-19 detection. Alternative approaches such as representation-based classification [collaborative or sparse representation (SR)] might provide satisfactory performance with limited size data sets, but they generally fall short in performance or speed compared to the neural network (NN)-based methods. To address this deficiency, convolution support estimation network (CSEN) has recently been proposed as a bridge between representation-based and NN approaches by providing a noniterative real-time mapping from query sample to ideally SR coefficient support, which is critical information for class decision in representation-based techniques. The main premises of this study can be summarized as follows: 1) A benchmark X-ray data set, namely QaTa-Cov19, containing over 6200 X-ray images is created. The data set covering 462 X-ray images from COVID-19 patients along with three other classes; bacterial pneumonia, viral pneumonia, and normal. 2) The proposed CSEN-based classification scheme equipped with feature extraction from state-of-the-art deep NN solution for X-ray images, CheXNet, achieves over 98% sensitivity and over 95% specificity for COVID-19 recognition directly from raw X-ray images when the average performance of 5-fold cross validation over QaTa-Cov19 data set is calculated. 3) Having such an elegant COVID-19 assistive diagnosis performance, this study further provides evidence that COVID-19 induces a unique pattern in X-rays that can be discriminated with high accuracy.

## I. Introduction

Coronavirus disease 2019 (COVID-19) has been declared as a pandemic by the World Health Organization (WHO) a few months after its first appearance. It has infected more than 70 million people, caused a few million causalities, and has so far paralyzed mobility all around the world. The spreading rate of COVID-19 is so high that the number of cases is expected to be doubled every three days if the social distancing is not strictly observed to slow this accretion [1]. Roughly around half of the COVID-19 positive patients also exhibit a comorbidity [2], making it difficult to differentiate COVID-19 from other lung diseases. Automated and accurate COVID-19 diagnosis is critical for both saving lives and preventing its rapid spread in the community. Currently, reverse transcription-polymerase chain reaction (RT-PCR) and computed tomography (CT) are the common diagnostic techniques used today. RT-PCR results are ready at the earliest 24 h for critical cases and generally take several days to conclude a decision [3]. CT may be an alternative at initial presentation; however, it is expensive and not easily accessible [4]. The most common tool that medical experts use for both diagnostic and monitoring the course of the disease is X-ray imaging. Compared to RT-PCR or CT test, having an X-ray image is an extremely low cost and a fast process, usually taking only a few seconds. Recently, WHO reported that even RT-PCR may give false results in COVID-19 cases due to several reasons such as poor quality specimen from the patient, inappropriate processing of the specimen, taking the specimen at an early or late stage of the disease [5]. For this reason, X-ray imaging has a great potential to be an alternative technological tool to be used along with the other tests for an accurate diagnosis.

**Fig. 1.**
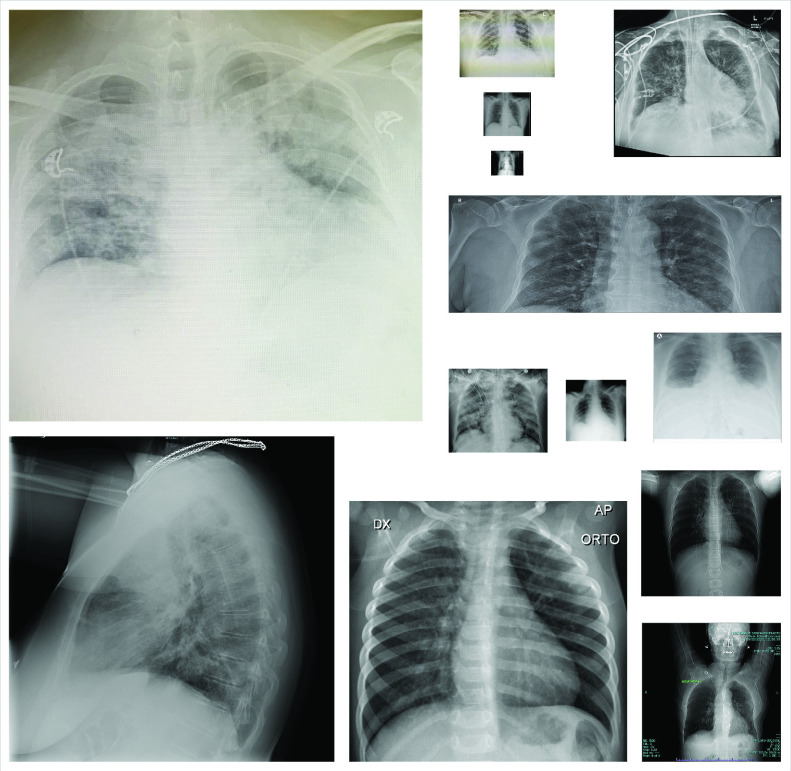
Sample COVID-19 X-ray images from QaTa-Cov19.

**Fig. 2.**
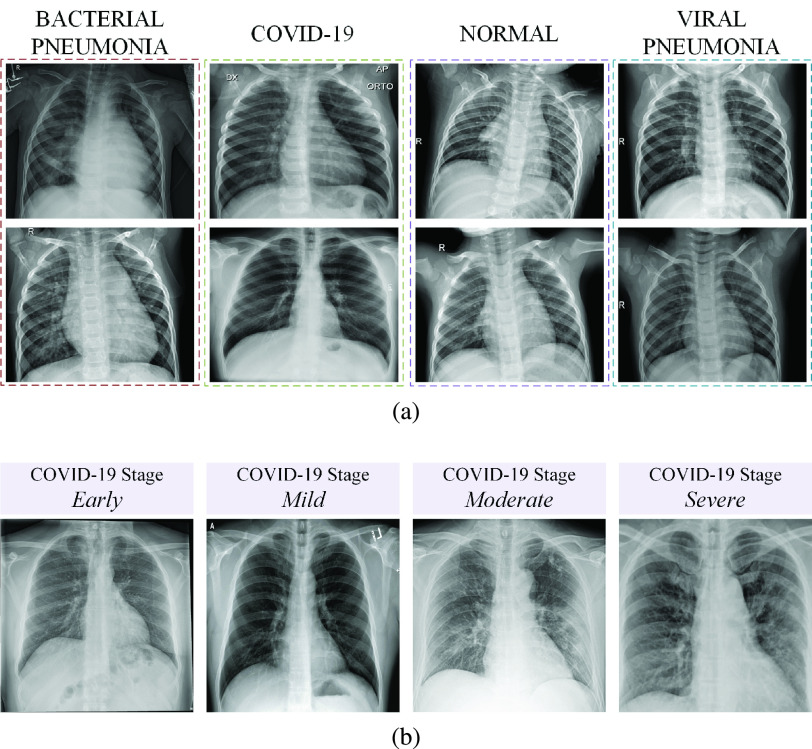
Sample QaTa-Cov19 X-ray images. (a) X-ray images from different classes. (b) X-ray images from the COVID-19 patients who are in the different stages.

In this study, we aim to differentiate X-ray images of COVID-19 patients among other classes; bacterial pneumonia, viral pneumonia, and normal. For this work, a benchmark COVID-19 X-ray data set, Qata-Cov19 (**Qa**tar University and **Ta**mpere University **COV**ID-**19** Data set) that contains 462 X-ray images from COVID-19 patients was collected. The images in the data set are different in quality, resolution, and SNR levels as shown in Fig. 1. QaTa-Cov19 also contains many X-ray images from the COVID-19 patients who are in the early stages; therefore, their X-ray images show mild or no-sign of COVID-19 infestation by the naked eye.^1^ Some sample images are shown in Fig. 2(b). Another fact that makes the diagnosis far more challenging is that interclass similarity can be very high for many X-ray images as some samples are shown in Fig. 2(a). Against such high interclass similarities and intraclass variations, in this study, we aim for a high robustness level.^1^The statements belong to the medical doctors whose names are listed in the Acknowledgment section.

In numerous classification tasks, deep learning techniques have been shown to achieve state-of-the-art performance in terms of both recognition accuracy and their parallelizable computing structures which play an important role, especially in real-time applications. Despite their advantages, in order to achieve the desired performance level in a deep model, proper training over a massive training data set is usually needed. Nevertheless, this is unfortunately unfeasible for this problem since the available data is still rather limited.

An alternative supervised approach, which requires a limited number of training samples to achieve satisfactory classification accuracy is representation-based classification [6]–[8]. In representation-based classification systems, a dictionary, the columns of which consist of the training samples that are stacked in such a way that a subset of them corresponding to a class, is predefined. A test sample is expected to be a linear combination of all points from the same class as the test sample. Therefore, given a predefined dictionary matrix, $\mathbf {D}$ and a test sample $\mathbf {y}$, we expect the solution $\mathbf {\hat {x}}$ from $\mathbf {y} = \mathbf {D x}$, carry enough information about the class of $\mathbf {y}$. Overall, in this study, we draw a convolutional support estimation network (CSEN) [9] -based solution pipeline, which fuses the representation-based classification scheme into a neural network (NN) body.

The rest of this article is organized as follows. In Section II, notations and mathematical preliminaries are given with emphasis on sparse representation (SR) and sparse support estimation (SE). Then in Section III, a literature review on deep learning models over X-ray images and representation-based classification is presented. The proposed CSEN-based COVID-19 recognition system is introduced in Section IV along with two recent alternative approaches that are used as the competing methods. The data collection is also explained in this section. Experimental setup and the main results are provided in Section V. Finally, Section VII concludes this article and suggests topics for future research.

## II. Preliminaries and Mathematical Notations

### A. Notations

In this study, the $\ell _{p}$-norm of a vector $\mathbf {x} \in \mathbb {R}^{n}$ is defined as $\left \|{ \mathbf {x} }\right \|_{\ell _{p}^{n}} = \left ({\sum _{i=1}^{n} \left \vert{ x_{i} }\right \vert ^{p} }\right)^{1/p}$ for $p \geq 1$. On the other hand, the $\ell _{0}$-norm of the vector $\mathbf {x} \in \mathbb {R}^{n}$ is defined as $\left \|{ \mathbf {x} }\right \|_{\ell _{0}^{n}} = \lim _{p \to 0} \sum _{i=1}^{n} \left \vert{ x_{i} }\right \vert ^{p} = \# \{ j: x_{j} \neq 0 \}$ and the $\ell _{\infty }$-norm is defined as $\left \|{ \mathbf {x} }\right \|_{\ell _{\infty }^{n}} = \max _{i=1,\ldots,n} \left ({\left |{ x_{i} }\right | }\right)$. A signal $\mathbf {s}$ is called strictly $k$-sparse if $\left \|{ \mathbf {x} }\right \|_{0} \leq k$. Sparse support set or simply support set, $\Lambda \subset \{1,2,3,\ldots,n \} $ of sparse signal $\mathbf {x}$ can be defined as the set of nonzero coefficients’ location, i.e., $\Lambda:= \left \{{ i: x_{i} \neq 0 }\right \}$.

### B. Sparse Signal Representation

SR of a signal $\mathbf {s} \in \mathbb {R}^{d} $ in a predefined set of waveforms, $\boldsymbol {\Phi } \in \mathbb {R}^{ d\times n}$, can be defined as representing $\mathbf {s}$ as a linear combination of only a small subset of atoms in the dictionary $\boldsymbol {\Phi }$, i.e., $\mathbf {s} = \boldsymbol {\Phi }\mathbf {x}$. Defining these sets, which dates back to Fourier’s pioneering work [10], has been excessively studied in the literature. In the early approaches, these sets of waveforms have been selected as a collection of linearly independent and generally orthogonal waveforms (which are called a complete dictionary or basis, i.e., $d=n$) such as Fourier transform, DCT, and wavelet transform, until the pioneering work of Mallat [11] on overcomplete dictionaries ( $n \gg d$). In the last decade, interest in SR research increased tremendously. Their wide range of applications includes denoising [12], classification [13], anomaly detection [14], [15], deep learning [16], and compressive sensing (CS) [17], [18].

With a possible dimensional reduction that can be satisfied via a compression matrix $\mathbf {A} \in \mathbb {R}^{m \times d} $ ( $m \ll d$), sample can be obtained from $\mathbf {s}$

\begin{equation*} \mathbf {y} = \mathbf {A} \mathbf {s} = \mathbf {A}\boldsymbol{\Phi } \mathbf {x} =\mathbf { D} \mathbf {x} \tag{1}\end{equation*}

where $\mathbf {D} \in \mathbb {R}^{m \times n}$ can be called the equivalent dictionary. Because (1) describes an underdetermined system of linear equations, finding the representation coefficient vector $\mathbf {x}$ requires at least one more constraint to have a unique solution. Using the prior information about sparsity, the following representation:

\begin{equation*} \min _{\mathbf {x}}~\left \|{ \mathbf {x }}\right \|_{0}~\text {s.t.}~\mathbf {D} \mathbf {x} = \mathbf {y} \tag{2}\end{equation*}

which is also an SR of $\mathbf {x}$ has a unique solution provided that $\mathbf {x}$ is strictly sparse and $\mathbf {D}$ satisfies some required properties [19]. For instance, if $\left \|{ \mathbf {x} }\right \|_{0} = k $, the minimum number of linearly independent columns of $\mathbf {D}$, $\text {spark}(\mathbf {D})$, should be greater than 2 k, i.e., $\text {spark}(\mathbf {D}) \geq 2\textrm {k}$ in order to not to have $\mathbf {D}\mathbf {x'} = \mathbf {D}\mathbf {x''} $ for distinct $k$-sparse signals, $\mathbf {x'}$ and $\mathbf {x''}$ [19]. However, the optimization problem in (2) is a NP-hard. Fortunately, the following relaxation:

\begin{equation*} \min _{\mathbf {x}}~\left \|{ \mathbf {x }}\right \|_{1}~\text {s.t.}~\mathbf {D} \mathbf {x} = \mathbf {y} \tag{3}\end{equation*}

produces exactly the same solution as that of (2) provided that $\mathbf {D}$ obeys some criteria: the equivalence of $\ell _{0}$– $\ell _{1}$ minimization problems can be guaranteed when $\mathbf {D}$ satisfies a notation of null space property (NSP) [20], [21] not only for exact sparse signals but approximately sparse signals. Furthermore, the query sample $\mathbf {y}$ can be corrupted with an additive noise pattern. In this case, the equality constraint in (3) can be further relaxed such as in the basis pursuit denoising (BPDN) [22]: $\min _{\mathbf {x}} \left \|{ \mathbf {x} }\right \|\,\,\text {s.t.}\,\,\left \|{ \mathbf {y} - \mathbf {Dx} }\right \| \leq \epsilon $, where $\epsilon $ is a small constant that depends on the noise level. In this case, a stronger property which is known as restricted isometry property (RIP) [23], [24] is frequently used which both cover conditions satisfying exact recovery of BP and stable recovery of BPDN, e.g., exact recovery of $\mathbf {x}$ from (3) is possible when $\mathbf {D}$ has RIP and $m > k (\log (n/k))$.

We may refer to the sparse SE problem as finding the indices a set, $\Lambda $, of nonzero elements of $\mathbf {x}$ [25], [26]. Indeed, in many applications, SE can be more important than finding the magnitude and sign of $\mathbf {x}$ as well as $\Lambda $, which refers to the sparse signal recovery (SSR) via a recovery technique, such as (3). For example, in a sparse representation-based classification (SRC) system, a query sample $\mathbf {y}$ can be represented with sparse coefficient vector, $\mathbf {x}$, in the dictionary, $\mathbf {D}$ in such a way that when we recover this representation coefficient from $\mathbf {y} = \mathbf {D} \mathbf {x}$, the solution vector $\mathbf {\hat {x}}$ is expected to have a significant number of nonzero coefficients coming from the particular locations corresponding to the class of $\mathbf {y}$.

Readers are referred to [9] for a more detailed literature review on SE and its applications. In the sequel, we briefly summarize the building blocks of the proposed approach.

## III. Background and Prior Art

### A. CheXNet

In the proposed approach, we first use the pretrained deep network, CheXNet, to extract discriminative features from raw X-ray images. CheXNet was developed for pneumonia detection from the chest X-ray images [27]. In [27], it was claimed that CheXNet can perform even better than expert radiologists in the pneumonia detection problem. This deep NN design is based on the previously proposed DenseNet [28] that consists of 121 layers. It is first pretrained over ImageNet data set [29] and performed transfer learning over 112120 frontal-view chest X-ray images in the ChestX-ray14 data set [30].

### B. Representation-Based Classification

Consider we are given a test sample $\mathbf {y}$, which represents either the extracted features, $\mathbf {s}$, or their dimensionally reduced version, i.e., $\mathbf {y} = \mathbf {A} \mathbf {s}$. In developing the dictionary, training samples are stacked in the dictionary $\mathbf {D}$ with particular locations in such a way that the optimal support for a given query $\mathbf {y}$ should be the set of all points coming from the same class as $\mathbf {y}$. Therefore, a solution vector, $\mathbf {\hat {x}}$ of $\mathbf {y} = \mathbf {D} \mathbf {x}$ is supposed to have enough information, i.e., the sparse support should be the set of location indices of the training sample from the same class as $\mathbf {y}$. This strategy is generally known as representation-based classification. However, a typical solution $\mathbf {\hat {x}}$ of $\mathbf {y} = \mathbf {D} \mathbf {x}$ is not necessarily a sparse one especially when its size grows with more training samples, which results in a highly underdetermined system of linear equations. Fortunately, if one estimates the representation coefficient vector with a sparse recovery design such as $\ell _{1}$-minimization as in (3), we can expect that the important nonzero entries of the solution, $\mathbf {\hat {x}}$, are grouped in the particular locations that correspond to the locations of the training samples from the same class as $\mathbf {y}$. This can be a typical example of scenarios where SE can be more valuable than the magnitudes and sign recovery as explained in Section II-B.

For instance, Wright *et al.* [8] proposed a systematic way of determining the identity of face images using $\ell _{1}$-minimization. The authors develop a three-step classification technique that includes: (i) normalization of all the atoms in $\mathbf {D}$ and $\mathbf {y}$ to have unit $\ell _{2}$-norm; (ii) estimating the representation coefficient vector via sparse recovery, i.e., $\hat {\mathbf {x}} = \arg \min _{\mathbf {x}} \left \|{\mathbf { x} }\right \|_{1}\,\,\text {s.t.}\left \|{ \mathbf {y} - \mathbf {D} \mathbf {x} }\right \|_{2} $; and (iii) finding the residuals corresponding to each class via $\mathbf {e_{i}} = \left \|{ \mathbf {y} - \mathbf {D_{i}} \mathbf {\hat {x}_{i}} }\right \|_{2}$, where $\mathbf {\hat {x}_{i}}$ is the group of the estimated coefficients, $\mathbf {\hat {x}}$, that correspond to class $i$.

This technique, which is known as SRC, and its variants have been applied to a wide range of applications in the literature [31], [32], e.g., human action recognition [33], and hyperspectral image classification [34], to name a few. Despite the good recognition accuracy performance of SRC systems, their main drawbacks is the fact that their sparse recovery algorithms (e.g., $\ell _{1}$-minimization) are iterative methods and computationally costly, rendering them infeasible in real-time applications. Later, the authors of [6] introduced collaborative representation-based classification (CRC), which is similar to SRC except for the use of traditional $\ell _{2}$-minimization in the second step; $\mathbf {\hat {x} }= \arg \min _{\mathbf {x}} \left \{{ \left \|{ \mathbf {y }- \mathbf {D}\mathbf {x }}\right \|_{2}^{2} + \lambda \left \|{ \mathbf {x} }\right \|_{2}^{2} }\right \}$. Thus, CRC does not require an iterative solution to obtain representation coefficient thanks to that $\ell _{2}$-minimization has a closed form solution, $\hat {\mathbf {x}} = \left ({\mathbf {D}^{\text {T}} \mathbf {D} + \lambda \mathbf {I}_{n \times n} }\right)^{-1} \mathbf {D}^{\text {T}} \mathbf { y}$. Although, the sparsity in $\mathbf {\hat {x}}$ cannot be guaranteed, it has often been reported to achieve a comparable classification performance, especially in small-size training data sets.

## IV. Proposed Approach

For a computer-aided COVID-19 recognition system design, our primary objective is to achieve the highest sensitivity possible in the diagnosis of COVID-19 induced pneumonia with an acceptable false-alarm rate (e.g., specificity $\gt 95\%$). In particular, the misdiagnosis of a COVID-19 X-ray image as a normal case should be minimized whilst a small number of false negatives (FNs) is tolerable.

Our interest in representation-based classification is that they perform well in classification tasks even in the cases where training data is scarce. As mentioned, the two well-known representation-based classification methodologies are SRC [7] and CRC [6]. Among them, SRC provides slightly improved accuracy by solving an SR problem, i.e., producing a sparse solution $\mathbf {\hat {x}}$ from $\mathbf {y} = \mathbf {D x}$. Then, the location of the nonzero elements of $\mathbf {\hat {x}}$, which is also known as support set, provides the class information of the query $\mathbf {y}$. Despite improved recognition accuracy, SRC solutions are iterative solutions and can be computationally demanding compared to CRC. In a recent work [9], a compact NN design that can be considered as a bridge between NN-based and representation-based methodologies was proposed. The so-called CSEN uses a predefined dictionary and learns a direct mapping using moderate/low size training set, which maps query samples, $\mathbf {y}$, directly to the support set of representation coefficients, $\mathbf {x}$ (as it should be purely sparse in the ideal case).

**Fig. 3.**
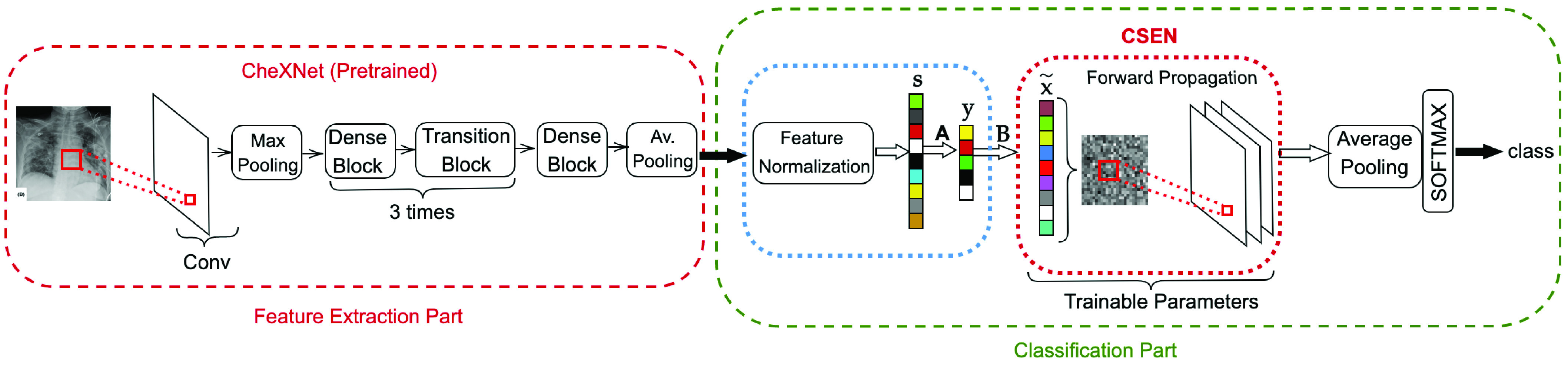
Proposed approach for Covid recognition from X-ray images. The proposed convolution support estimator network (CSEN) which can be trained from a moderate size training set. The pipeline employs the pretrained deep NN for feature extraction. $\mathbf {A}$ is the dimensional reduction (PCA) matrix, the coarse estimation of representation coefficient (sparse in ideal case), $\hat {\mathbf {x}}$ is obtained via the denoiser matrix, $\mathbf {B} = \left ({\mathbf {D}^{T} \mathbf {D} + \lambda \mathbf {I} }\right)^{-1} \mathbf {D}^{T}$, where $\mathbf {D}=\mathbf {A}\boldsymbol{\Phi }$ and $\boldsymbol{\Phi }$ is the predefined dictionary matrix of training samples (before dimensional reduction).

In this study, to address the data scarcity limitations in COVID-19 diagnosis from X-ray images we propose a CSEN-based approach. Since a relatively larger set of COVID-19 X-ray images ever compiled is used in this study, the proposed approach can be evaluated rigorously against a high level of diversity to obtain a reliable analysis. The general pipeline of the proposed CSEN-based recognition scheme is illustrated in Fig. 3. In order to obtain highly discriminative features, we use the recently proposed CheXNet [27], which is the fine-tuned version of 121 layer Dense Convolutional Network (DenseNet-121) [28] by using over $100\thinspace 000$ frontal view X-ray images form 14 classes. Having the pretrained CheXNet for feature extraction, we develop two different strategies to obtain the classes of query X-ray images: 1) using CRC with proper preprocessing; 2) a slightly modified version of our recently proposed convolution support estimator (CSEN) models. In the sequel, both techniques will be explained in detail as well as alternative solutions.

### A. Benchmark Data Set: QaTa-Cov19

Accordingly, there are several recent works [35]–[38] that have been proposed for COVID-19 detection/classification from X-ray images. However, they use a rather small data set (the largest containing only a few hundreds of X-ray images), with only a few COVID-19 samples. This makes it difficult to generalize their results in practice. To address this deficiency and provide reliable results, in this study the researchers of **Qa**tar University and **Ta**mpere University have compiled a bechmark **Cov**id-**19** data set, called QaTa-Cov19. Compared to the earlier benchmark data set created in this domain, such as COVID Chestxray Data set [39] or COVID-19 DATA SET [40], QaTa-Cov19 has the following unique benchmarking properties. First, it is a larger data set, not only in terms of the number of images (more than 6200 images) but its versatility, i.e., QaTa-Cov19 contains additional major pneumonia categories, such as viral and bacterial, along with the control (normal) class. Moreover, this is a diverse data set encapsulating X-ray images from several countries (e.g., Italy, Spain, China, etc.) produced by different X-ray machines.

**Fig. 4.**
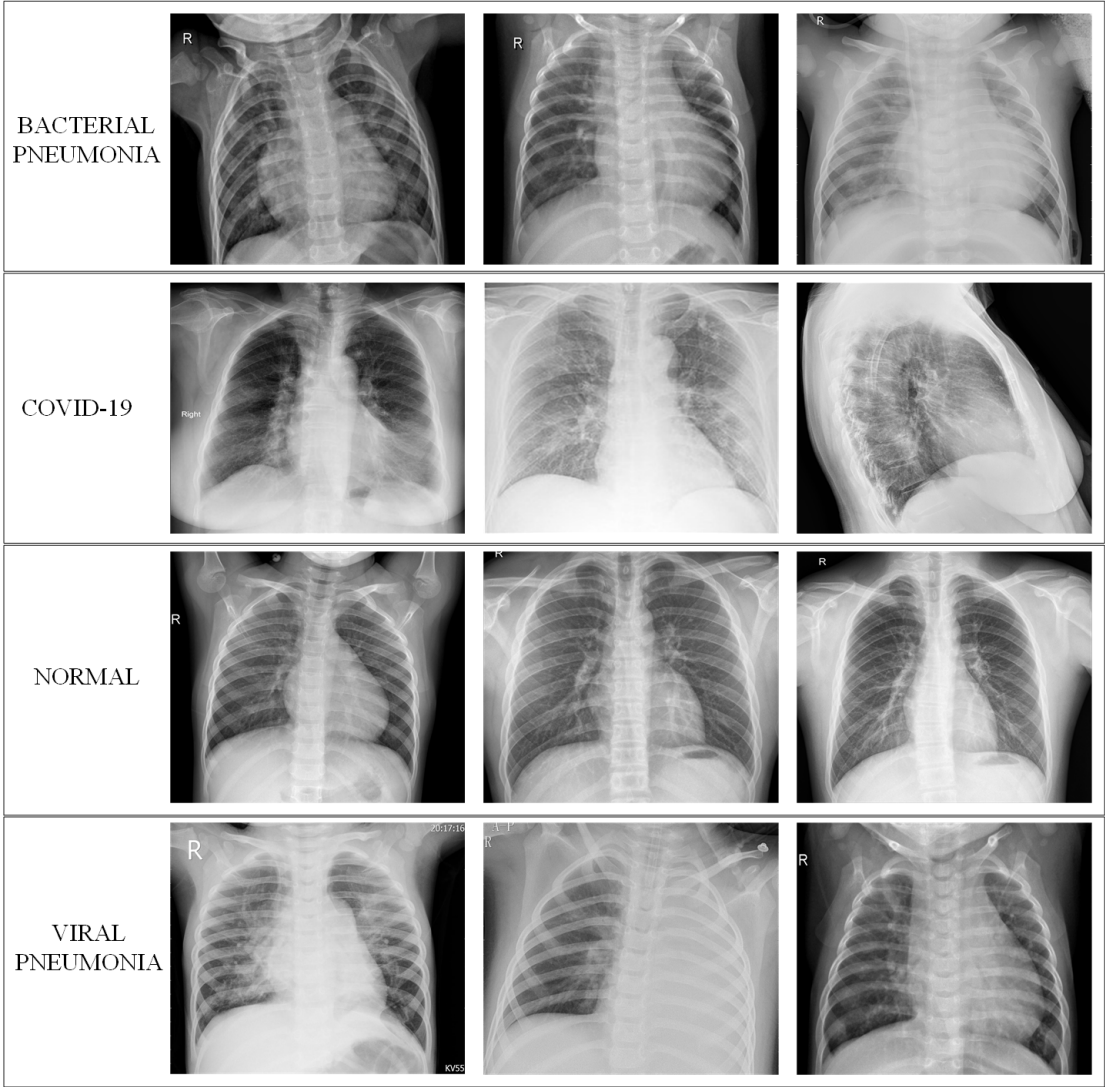
Samples from the benchmark QU-Chest data set.

COVID-19 chest X-ray images were gathered from different publicly available but scattered image sources. However, the major sources of COVID-19 images are Italian Society of Medical and Interventional Radiology (SIRM) COVID-19 Database [40], Radiopaedia [41], Chest Imaging (Spain) at thread reader [42] and online articles and news portals [43]. The authors have carried out the task of collecting and indexing the X-ray images for COVID-19 positive cases reported in the published and preprint articles from China, South Korea, USA, Taiwan, Spain, and Italy, as well as online news-portals (up to 20th April 2020). Therefore, these X-ray images represent different age groups, gender, ethnicity, and country. Negative Covid19 cases were normal, viral, and bacterial pneumonia chest X-ray images and collected from the Kaggle chest X-ray database. Kaggle chest X-ray database contains 5863 chest X-ray images of normal, viral, and bacterial pneumonia with varying resolutions [44]. Out of these 5863 chest X-ray images, 1583 images are normal images and the remaining are bacterial and viral pneumonia images. Sample X-ray images from QaTa-Cov19 data set are shown in Fig. 4.

### B. Feature Extraction

With their outstanding performance in image classification along with other inference tasks, deep NNs became a dominant paradigm. However, these techniques usually necessitate a large number of training samples (e.g., several hundred-thousand to millions depending on the network size) to achieve an adequate generalization capability. Albeit, we can still leverage their power by finding properly pretrained models for similar problems. To this end, we use a state-of-the-art pneumonia detection network, CheXNet, whose details are summarized in Section III-A. With the pretrained model, we extract 1024-long vectors, right after the last average pooling layer. After data normalization (zero mean and unit variance), we obtain a feature vector $\mathbf {s} \in \mathbb {R}^{d=1024}$.

A dimensionality reduction PCA is applied to $\mathbf {s}$ in order to get the query sample, $\mathbf {y} = \mathbf {A} \mathbf {s} \in \mathbb {R}^{m}$, where $\mathbf {A} \in \mathbb {R}^{m \times d} $ is PCA matrix ( $m< d$).

### C. Proposed CSEN-Based Classification

Considering the limited number of training data in our COVID-19 data set, a representation-based classification can be applied hereafter to obtain the class of $\mathbf {y}$ using the dictionary $\boldsymbol {\Phi }$ (in the form of $\mathbf {D} = \mathbf {A \Phi }$), whose columns are stacked training samples with class-specific locations.

As discussed earlier, SRC is an SE problem which is expected to be an easier task than an SSR problem. On the other hand, even if the exact signal recovery is not possible in noisy cases or in cases where $\mathbf {\hat {x}}$ is not exactly but approximately sparse (which is the case almost all the time in dictionary-based classification problems), it is still possible to recover the support set exactly [25], [38], [45], [46] or partially [46]–[48]. However, many works in the literature dealing with SE problems tend to first apply a sparse recovery technique on $\mathbf {y}$ to first get $\mathbf {\hat {x}}$, then use simple thresholding over $\mathbf {\hat {x}}$ to obtain a sparse SE, $\hat {\Lambda }$. However, SSR techniques such as $\ell _{1}$-minimization are rather slow and their performance varies from one SRR tool to another [9]. In our previous work [9], we proposed an alternative solution for this iterative sparse recovery approach which aims to learn a direct mapping from a test sample $\mathbf {y}$ to the corresponding support set $\hat {\Lambda }$. Along with the speed and stability compared to conventional SSR-based techniques and recent deep learning-based SSR solutions, CSEN has the crucial advantage of having a compact design that can achieve a good performance level even over scarce training data.

Mathematically speaking, an ideal CSEN is supposed to yield a binary mask $\mathbf {v} \in \left \{{ 0,1 }\right \}^{n}$

\begin{equation*} {v_{i} =} 1 ~ \text {if $i \in \Lambda $}\tag{4}\end{equation*}

which indicates the true support, i.e., ${\Lambda } = \left \{{ i \in \left \{{ 1,2,\ldots,n}\right \}: {v}_{i} =1 }\right \}$. In order to approximate this ideal case, a CSEN network, $\mathcal {P} \left ({\mathbf {y}, \mathbf {D} }\right)$ produces a probability vector $\mathbf {p} $ which returns a measure about the probability of each index being in ${\Lambda }$ such that $p_{i} \in \left [{ 0,1 }\right]$. Having the estimated probability map, estimating the support can easily be done via $\hat {\Lambda } = \left \{{ i \in \left \{{ 1,2,\ldots,n}\right \}: p_{i} > \tau }\right \}$, by thresholding $\mathbf {p}$ with $\tau $ where $\tau $ is a fixed threshold.

A CSEN is composed of fully convolutional layers, and as input it takes a proxy, $\mathbf {\tilde {x}}$, of sparse coefficient vector, which is a coarse estimation of $\mathbf {x}$, i.e., $\left ({\mathbf {D}^{T} \mathbf {D} + \lambda \mathbf {I} }\right)^{-1} \mathbf {D}^{T}\mathbf {y}$ or simply $\mathbf {\tilde {x}}=\mathbf {D}^{T}\mathbf {y}$. Then, it yields the aforementioned probability like vector $\mathbf {p} $ via fully convolutional layers. Using such a proxy of $\mathbf {x}$, instead of making inference directly on $\mathbf {y}$ has also studied in a few more recent studies. For instance, in [49] and [50], the authors proposed reconstruction-free image classification from compressively sensed images. Alternatively, one may design a network to learn proxy $\mathbf {\tilde {x}}$ by fully connected dense layers [49]. However, it increases the computational complexity and may result in an even over-fitting problem with scarce training data [9].

**Fig. 5.**
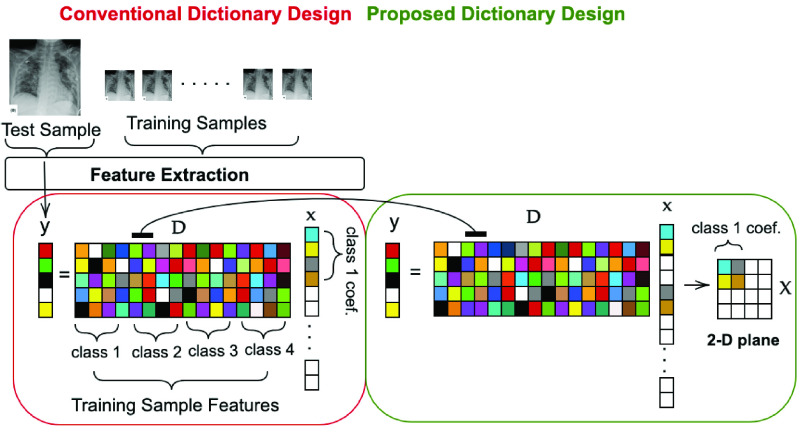
Illustration of proposed dictionary design versus conventional design in representation-based classifiers.

The input vector $\mathbf {\tilde {x}}$ is reshaped to have a 2-D plane representation in order to use it with 2-D convolutional layers. This transformation is performed via reordering the indices of the atoms in such a way that the nonzero elements of the representation vector $\mathbf {x}$ for a specific class come together in the 2-D plane. A representative illustration of the proposed dictionary design compared to the traditional one is shown in Fig. 5.

Hereafter, the proxy $\mathbf {\tilde {x}}$ is convolved with the weight kernels, connecting the input with the next layer with $N_{l}$ filters to yield the inputs of the next layer, with the biases $\mathbf {b_{1}}$ as follows:

\begin{equation*} \mathbf {f_{1}} = \left \{{S_{1}\left ({\text {ReLu}\left ({\mathbf {b_{1}^{i}} + \mathbf {w_{1}^{i}} * \tilde {\mathbf {x}}}\right)}\right)}\right \}_{i=1}^{N_{1}}\tag{5}\end{equation*}

where $\mathbf {b_{1}}$ is the weight bias, $S_{1}(.)$ is either identity or sub-sampling operator predefined according to network structure and $\text {ReLu}(x) = \max (0, x)$. For other layers, i.e., $l>2$, the $k{\text {th}}$ feature map of layer $l$ is defined as

\begin{equation*} \mathbf {f_{l}^{k}} = S_{l} \left ({\text {ReLu} \left ({\mathbf {b_{l}^{k}} + \sum _{i}^{N_{l-1}} \mathbf {w_{l}^{ik}} * \mathbf {f_{l-1}^{i}} }\right) }\right)\tag{6}\end{equation*}

where $S_{l}(.)$ is either identity operator or one the operations from down- and up-sampling and $N_{l}$ is the number of feature maps in $l{\text {th}}$ layer. Therefore, the trainable parameters of CSEN will be: $\mathbf {\Theta _{CSEN}}=\big \{ \{\mathbf {w}_{1}^{i}, \mathbf {b_{1}^{i}}\}_{i=1}^{N_{1}}, \{\mathbf {w}_{2}^{i}, \mathbf {b_{2}^{i}}\}_{i=1}^{N_{2}}, \ldots, \{\mathbf {w}_{L}^{i}, \mathbf {b_{L}^{i}}\}_{i=1}^{N_{L}}\big \}$ for an ${L}$ layer CSEN design.

In developing the dictionary that is to be used in the SRC, the training samples are stacked-in by grouping them according to their classes. Thus, instead of using traditional $\ell _{1}$-minimization formulation as in (3), the following group $\ell _{1}$-minimization formulation may result in increased classification accuracy:

\begin{equation*} \min _{\mathbf {x}} \left \{{ \left \|{ \mathbf {D}\mathbf {x}-\mathbf {y} }\right \|_{2}^{2} + \lambda \sum _{i=1}^{c}\left \|{\mathbf {x_{Gi}} }\right \|_{2} }\right \}\tag{7}\end{equation*}

where $\mathbf {x_{Gi}}$ is the group of coefficients from the $i{\text {th}}$ class. In this manner, one possible cost function for a SE network would be

\begin{equation*} E(\mathbf {x}) = \sum _{p} (\mathcal {P}_{\Theta }\left ({\mathbf {\tilde {x}} }\right)_{p}- v_{p})^{2} + \lambda \sum _{i=1}^{c}\left \|{\mathcal {P}_{\Theta }\left ({\mathbf {\tilde {x}} }\right)_{Gi} }\right \|_{2} \tag{8}\end{equation*}

where $\mathcal {P}_{\Theta }\left ({\mathbf {\tilde {x}} }\right)_{p}$ is network output at location $p$ and $v_{p}$ is the ground truth binary mask of the sparse code $\mathbf {x}$. Due to its high computational complexity, we approximate the cost function in (8) with a simpler average pooling layer after convolutional layer, which can produce directly the estimated class in our CSEN design. An illustration of proposed CSEN-based COVID-19 recognition is shown in Fig. 3.

### D. Competing Methods

This section summarizes the competing methods that are selected among numerous alternatives due to their superior performance levels obtained in similar problems. For fair comparative evaluations, all classification methods have the same input feature vectors fed to the proposed CSENs.

#### 1) Collaborative Representation-Based Classification:

**Fig. 6.**
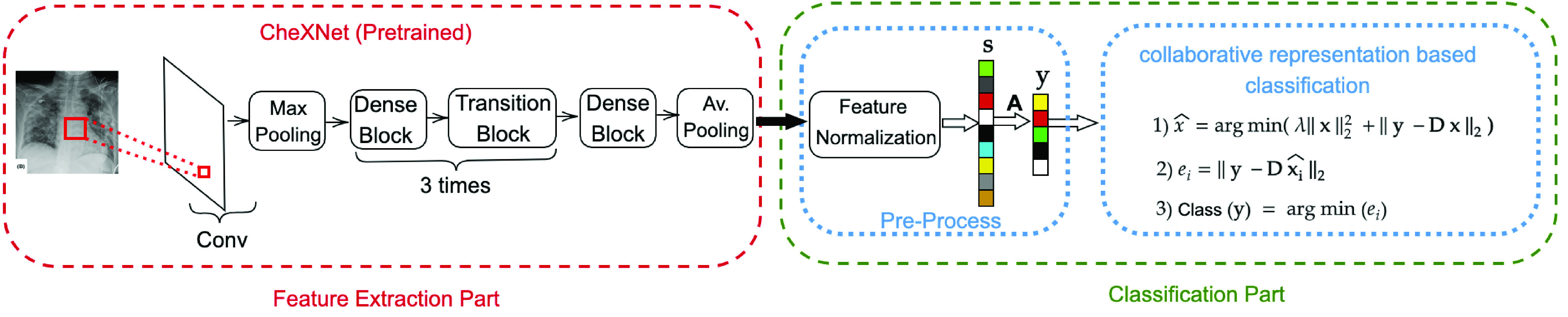
Baseline Approach I: CRC is fed by deep learning-based extracted features that are preprocessed.

As a possible competing technique to the proposed CSEN-based technique which is a hybrid method, CRC [6] is a direct and representation-based classification method that can be applied to this problem as shown in Fig. 6. It is a noniterative SE technique, that satisfies faster and comparable classification performance with SRC while it is more stable compared to existing iterative sparse recovery tools as it is shown in [9]. In the first step of CRC, the tradeoff parameter of the regularized least-square solution is set as $\lambda =2*10^{-12}$. In order to obtain the best possible $\lambda $, a grid search was made in the range $[10^{-15}, 10^{-1}]$ with a log scale.

#### 2) Multilayer Perceptron (MLP) Classification:

**Fig. 7.**
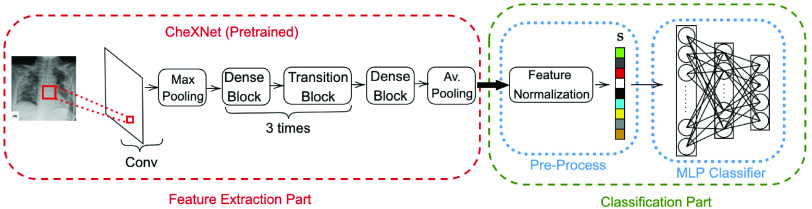
Baseline Approach II: A 5-layer MLP layer is used over the features of CheXNet.

**Fig. 8.**
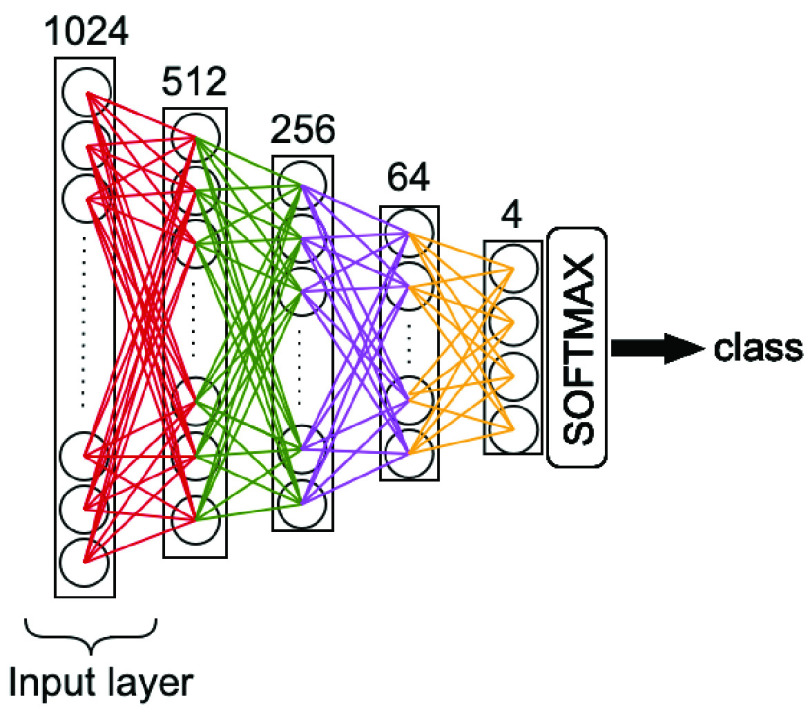
MLP configuration.

The proposed COVID-19 recognition pipeline can be modified by replacing CSEN or CRC part with another classifier. As one of the most-common classifiers, a 4-hidden layer multilayer perceptron (MLP) is used for this problem as shown in Fig. 7. For training, we used back-propagation (BP) with Adam optimization technique [51]. The network and training hyperparameters are as follows: learning rate, $\alpha = 10^{-4}$, and moment updates $\beta _{1} =0.9$, $\beta _{2} =0.999$, and 50 as the number of epochs. Fig. 8 illustrates the network configuration in detail. This network configuration has achieved the best performance among others (deeper and shallower) where deep configurations have suffered from *over-fitting* while the shallow ones exhibit an inferior learning performance.

#### 3) Support Vector Machines (SVMs):

For a multiclass problem, the first objective is to select the SVM topology for ensemble learning: one-versus-one or one-versus-all. In order to find the optimal topology and the hyperparameters (e.g., kernel type and its parameters) we first performed a grid-search with the following variations and setting: kernel function {linear, radial basis function (RBF)}, box constraint ( $C$ parameter) in the range $[{1, 10^{3}}]$ with a log scale, and kernel scale ( $\gamma $ for the RBF kernel) in the range $[10^{-4}, 10^{-2}]$ with a log scale.

#### 4) k-Nearest-Neighbor (k-NN):

Finally, we use a traditional approach, $k$-nearest neighbor ( $k$-NN) is used with PCA dimensionality reduction. In a similar fashion, the distance metric and the $k$-value are optimized by a prior grid-search. The following distance metrics are evaluated: City-block, Chebyshev, correlation, cosine, Euclidean, Hamming, Jaccard, Mahalanobis, Minkowski, standardized Euclidean, and Spearman metrics. The $k$-value is varied within the range of $[{1, 4416}]$ with a log scale.

## V. Experimental Results

### A. Experimental Setup

We have performed our experiments over the QaTa-Cov19 data set, which consists of normal and three pneumonia classes: bacterial, viral, and COVID-19. The proposed approach is evaluated using a stratified fivefold cross-validation (CV) scheme with a ratio of 80% for training and 20% for the test (unseen folds) splits, respectively.

Table II shows the number of X-ray images per class in the QaTa-Cov19 data set. Since the data set is unbalanced, we have applied data augmentation to the training set in order to balance the size of each class in the train set. Therefore, the X-ray images in viral and COVID-19 pneumonia and normal classes are augmented up to the same number as the bacterial pneumonia class in the train set. We use Image Data Generator by Keras to perform data augmentation by randomly rotating the X-ray images in a range of 10°, randomly shifting images both horizontally and vertically within the interval of $[-0.1, +0.1]$. In each CV fold, we use a total of 8832 and 1257 images in the train and test (unseen in the fold) sets, respectively.

**TABLE I:** Classification Performances of the Proposed CSEN and Competing Methods. The Best COVID-19 Recognition Rates Are Highlighted

		k-NN	SVM	MLP	CRC	ReconNet	CSEN1	CSEN2
Accuracy	Bacterial	0.777	0.780	0.763	0.820	0.765	0.793	0.794
	Viral	0.801	0.787	0.765	0.827	0.785	0.805	0.803
	Normal	0.903	0.934	0.933	0.928	0.918	0.926	0.927
	COVID-19	0.950	0.945	0.949	0.955	0.936	0.955	0.959
TN	Bacterial	3166	3219	3114	3063	3180	3177	3173
	Viral	4123	3965	3923	4385	4005	4109	4091
	Normal	4253	4444	4442	4380	4364	4388	4396
	COVID-19	5525	5489	5522	5554	5435	5548	5572
TP	Bacterial	1720	1687	1680	2091	1629	1810	1818
	Viral	909	979	884	816	928	954	959
	Normal	1420	1427	1421	1456	1407	1431	1428
	COVID-19	446	452	444	447	448	455	455
FP	Bacterial	360	307	412	463	346	349	353
	Viral	678	836	878	416	796	692	710
	Normal	454	263	265	327	343	319	311
	COVID-19	299	335	302	270	389	276	252
FN	Bacterial	1040	1073	1080	669	1131	950	942
	Viral	576	506	601	669	557	531	526
	Normal	159	152	158	123	172	148	151
	COVID-19	16	10	18	15	14	7	7
Sensitivity	Bacterial	0.623	0.611	0.609	0.758	0.590	0.656	0.659
	Viral	0.612	0.660	0.595	0.550	0.625	0.642	0.646
	Normal	0.899	0.904	0.900	0.922	0.891	0.906	0.904
	COVID-19	0.965	0.978	0.961	0.968	0.970	0.985	0.985
Specificity	Bacterial	0.898	0.913	0.883	0.869	0.902	0.901	0.900
	Viral	0.859	0.826	0.817	0.913	0.834	0.856	.852
	Normal	0.904	0.944	0.944	0.931	0.927	0.932	0.934
	COVID-19	0.949	0.943	0.948	0.954	0.933	0.953	0.957
F1-score	Bacterial	0.711	0.710	0.693	0.787	0.688	0.736	0.737
	Viral	0.592	0.593	0.545	0.601	0.578	0.609	0.608
	Normal	0.823	0.873	0.870	0.866	0.845	0.860	0.861
	COVID-19	0.740	0.724	0.735	0.758	0.690	0.763	0.778

**TABLE II:** Number of Images per Class and per-Fold Before and After Data Augmentation

Class	# of Samples	Training Samples	Augmented Training Samples	Test Samples
Bacterial Pneumonia	2760	2208	2208	552
Viral Pneumonia	1485	1188	2208	297
Normal	1579	1263	2208	316
COVID-19	462	370	2208	92
Total	6286	5029	8832	1257

The experimental evaluations of SVM, $k$-NN, and CRC are performed using MATLAB version 2019a, running on PC with Intel^®^ i7-8650U CPU and 32 GB system memory. On the other hand, MLP and CSEN methods are implemented using Tensorflow library [52] with Python on NVidia^®^ TITAN-X GPU card. For the CSEN training, ADAM optimizer [51] is used with the proposed default learning parameters: learning rate, $\alpha =10^{-3}$, and moment updates $\beta _{1} = 0.9$, $\beta _{2} = 0.999$ with only 15 back-propagation epochs. Neither grid-search nor any other parameter or configuration optimization was performed for CSEN.

### B. Experimental Results

The same network configurations are used for CSEN as in [9]. Accordingly, we use two compact CSEN designs: CSEN1 and CSEN2, respectively. The first CSEN network consists of only two hidden convolutional layers, the first layer has 48 neurons and the second has 24. ReLu activation function is used in the hidden layers and the filter size was $3\times 3$. On the other hand, CSEN2 uses max-pooling and has one additional hidden layer with 24 neurons to perform transposed-convolution. CSEN1 and CSEN2 are compared against the 6 competing methods under the same experimental setup.

For the dictionary construction in $\boldsymbol {\Phi }$ each CSEN design, 625 images for each class (from the augmented training samples per fold) are stacked in such way that the representation coefficient in the 2-D plane, $\mathbf {X}$ has $50\times 50$ size as shown in Fig. 5. The rest of the images in the training set are used to train each CSEN, i.e., 1583 samples from each class. We use PCA dimensional reduction matrix, $\mathbf {A}$ with the compression ratio, $\text {CR} = ({m}/{d}) = 0.5$. Therefore, we have $512 \times 2500$ equivalent dictionary, $\mathbf {D}$, and $2500 \times 512$ denoiser $\mathbf {B} = \left ({\mathbf {D}^{T} \mathbf {D} + \lambda \mathbf {I} }\right)^{-1} \mathbf {D}^{T}$ to obtain a coarse estimation of the representation (sparse in the ideal case) coefficients, $\mathbf {\tilde {x}} \in \mathbb {R}^{n = 2500}$. Hereafter, the CSEN networks are trained to obtain the class information of $\mathbf {y}$ from input $\mathbf {\tilde {x}}$ as illustrated in Fig. 3.

Due to the lack of other learning-based SE studies in the literature, we chose a deeper network compared to CSEN designs to investigate the role of network depth in this problem. ReconNet [53] was proposed as a noniterative deep learning solution to CS problem, i.e., $\mathbf { \hat {s} } \leftarrow \mathcal {P} \left ({\mathbf {y} }\right) $ and it is one of the state of the art in compressively sensed image recognition task. It consists of six fully convolutional layers and one dense layer in front of the convolutional ones, which act as the learned denoiser for the mapping from $\mathbf {y} \in \mathbb {R}^{m}$ to $\mathbf {\tilde {s}} \in \mathbb {R}^{d}$. Then, the convolutional layers are responsible for producing the reconstructed signal, $\mathbf {\hat {s}}$ from $\mathbf {\tilde {s}}$. Therefore, by replacing this dense layer with the denoiser matrix $\mathbf {B}$, this network can be used as a competing method.

Both CSEN and the modified ReconNet use $\mathbf {\tilde {x}}$ as an input, which is produced using an equivalent dictionary $\mathbf {D}$ and its pseudo-inverse matrix $\mathbf {B}$.

In designing the dictionary of the CRC system, all training samples are stacked in the dictionary, $\boldsymbol {\Phi }$, i.e., 2208 samples from each class. The same PCA matrix used in CSEN-based recognition, $\mathbf {A}$ is applied to features, $\mathbf {s} \in \mathbb {R}^{d=1024}$. Therefore, a dictionary $\mathbf {D}$ of size $512 \times 8832$ and the corresponding denoiser matrix $\mathbf {B}$ of size $8832 \times 512$ are used in the CRC framework.

Overall, the confusion matrix elements are formed as follows: true positive (TP): the number of correctly detected positive class members, true negative (TN): the number of correctly detected negative class samples, false positive (FP): the number of misclassified negative class members as positive, and FN: the number of misclassified positive class samples as negative (i.e., missed positive cases). Then, the standard performance evaluation metrics are defined as follows:

\begin{equation*} \text {Sensitivity} = \frac {\text {TP}}{\text {TP} + \text {FN}}\tag{9}\end{equation*}

where sensitivity (or Recall) is the rate of correctly detected positive samples in the positive class

\begin{equation*} \text {Specificity} = \frac {\text {TN}}{\text {TN} + \text {FP}}\tag{10}\end{equation*}

where specificity is the ratio of accurately detected negative class samples to all negative class

\begin{equation*} \text {Precision} = \frac {\text {TP}}{\text {TP} + \text {FP}}\tag{11}\end{equation*}

where precision is the rate of correctly classified positive class samples among all the members classified as a positive sample

\begin{equation*} \text {Accuracy} = \frac {\text {TP}+\text {TN}}{\text {TN} + \text {TP}+ \text {FP}+\text {FN}}\tag{12}\end{equation*}

where accuracy is the ratio of correctly classified elements among all the data

\begin{equation*} F (\beta) = \left ({1 + \beta ^{2} }\right) \frac { \left ({\text {Precision}+\text {Sensitivity} }\right) }{ \left ({\beta ^{2} *\text {Precision} }\right) + \text {Sensitivity}}\tag{13}\end{equation*}

where $F$-score is defined by the weighting parameter $\beta $. The $F1$-score is calculated with $\beta = 1$, which is the harmonic average of precision and sensitivity.

The classification performance of the proposed CSEN-based approach and the competing methods is presented in Table I. As can be easily observed from Table I, the proposed approaches surpass all competing methods in COVID-19 recognition performance by achieving 98.5% sensitivity, and over 95% specificity. As shown in Table III, compared to MLP and ReconNet, the proposed CSEN designs are very compact and computationally efficient. This is evident in Table IV where the computational complexity (measured as total computation, time over the 1257 test images) is reported.

**TABLE III:** Number of Network Parameters of Each Method

	MLP	CSEN1	CSEN2	ReconNet
# of trainable parameters	672,836	11,089	16,297	22,914

**TABLE IV:** Computation Times (Sec) of Each Method Over 1257 Test Images

	CRC (light)	CRC	CSEN1	CSEN2	ReconNet	MLP
Computation Time (in sec.)	13.4176	40.7878	0.2196	0.2272	0.2993	0.2935

**Fig. 9.**
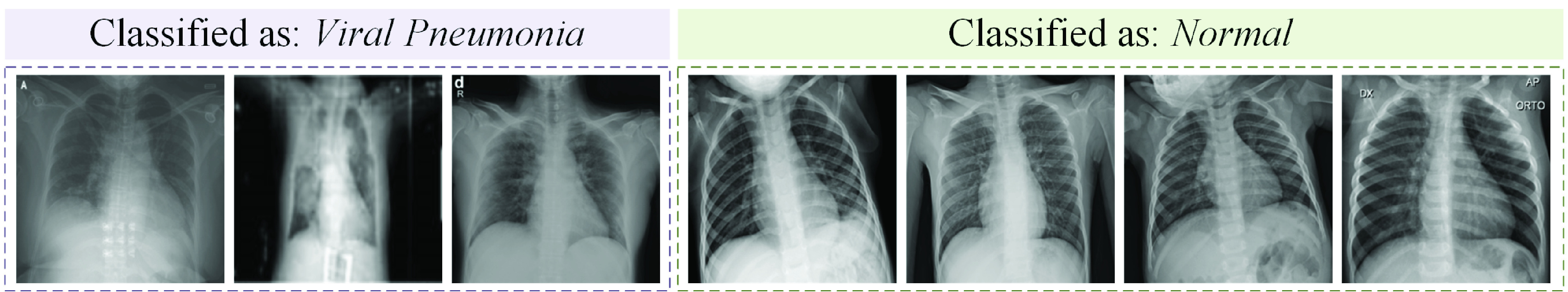
FNs of the proposed COVID-19 recognition scheme.

Finally, Table V presents the overall (cumulative) confusion matrix of the proposed CSEN-based COVID-19 recognition approach over the new QaTa-Cov19 data set. The most critical misclassifications are the false-positives, i.e., the misclassified COVID-19 X-ray images. The confusion matrix shows that the proposed approach has misclassified seven COVID-19 images (out of 462). The 3 out of 7 misclassifications are still in “viral pneumonia” category, which can be an expected confusion due to the viral nature of COVID-19. However, the other four cases are misclassified as “Normal” which is indeed a severe clinical misdiagnosis. A close look at these false-negatives in Fig. 9 reveals the fact that they are indeed very similar to normal images where typical COVID-19 patterns are hardly visible even by an expert’s naked eye. It is possible that these images come from patients who were in the very early stages of COVID-19.

**TABLE V:** Overall (Cumulative) Confusion Matrix of the Proposed Recognition Scheme

CSEN2	Predicted				
		Bacterial	Viral	Normal	COVID-19
Real	Bacterial	1818	636	180	126
	Viral	338	959	127	61
	Normal	15	71	1428	65
	COVID-19	0	3	4	455

## VI. Discussion

### A. CRC Versus CSEN

When compared against CRC in particular, CSEN-based classification has two advantages; computational efficiency and, a superior COVID-19 recognition performance. The computational efficiency comes from the fact that a larger size dictionary matrix (of the size of $512 \times 8832$) is used in CRC and hence, this requires more computations in terms of matrix-vector multiplications. Furthermore, saving the trainable parameters ( $\sim 16\text{k}$) and a light dictionary matrix coefficients ( $\sim 1280\text{k}$) in the test device is more memory efficient compared to saving coefficients ( $\sim 4521\text{k}$) of larger size dictionary used in CRC.

For further analysis, we also tested the CRC framework by using the light dictionary (of size $512 \times 2500$) used in CSEN-based recognition. We called it CRC (light), and as it can be seen in Table VI, the performance of CRC further reduced, and there was no significant improvement concerning the computational cost. When it comes to creating deeper convolutional layers instead of using CSEN designs, such as the modified ReconNet, the results presented in Table I shows us that compact CSEN structures are indeed preferable to achieve superior classification performances compared to deeper networks.

**TABLE VI:** Performance of CRC Algorithm When the Dictionary (Size of 625 per Class) That Is Used in CSEN Is Used

	CRC (Light)		
	Accuracy	Sensitivity	Specificity
Bacterial	0.8129	0.7464	0.8650
Viral	0.8163	0.5461	0.8998
Normal	0.9267	0.9170	0.9299
COVID-19	0.9564	0.9394	0.9578

### B. Compact Versus Deep CSENs

Representation-based classifications are known for providing satisfactory performance when it comes to limited size data sets. On the other hand, deep artificial NNs usually require a large training set to achieve a satisfactory generalization capability.

In a representation-based (dictionary) classification scheme when the dictionary size getting bigger (increase the number of training samples), the computational complexity of the method drastically increases. The proposed CSEN is an alternative approach to handle both moderate and scarce data sets via compact as possible NN structures for the dictionary-based classification.

Since there is no other learning-based SE method except CSEN in the literature, we chose ReconNet as a possible competing algorithm for this problem as explained in detail in Section V. ReconNet has six fully convolution layers. As an ablation study, we also add more hidden layers to proposed CSEN models to compare: CSEN3 and CSEN4 models were obtained by adding one and two hidden layers to CSEN2, respectively, after the transposed convolutional layer. Additional layers have 24 neurons, ReLu activation functions and filter size $3 \times 3$. As we can observe from Tables VII and VIII, the proposed compact designs, CSEN1 and CSEN2, both surpass deeper counterparts both in performance and the required number of parameters.

**TABLE VII:** Performance of Alternative Deeper Designs Compared to Compact CSENs

	Accuracy		Sensitivity		Specificity	
	CSEN3	CSEN4	CSEN3	CSEN4	CSEN3	CSEN4
Bacterial	0.793	0.792	0.651	0.653	0.904	0.900
Viral	0.808	0.805	0.642	0.638	0.859	0.856
Normal	0.922	0.921	0.907	0.899	0.927	0.928
Covid-19	0.954	0.954	0.990	0.987	0.951	0.952

**TABLE VIII:** Number of Network Parameters of Competing SE Networks

	CSEN1	CSEN2	CSEN3	ReconNet	CSEN 4
# of trainable parameters	11,089	16,297	21,505	22,914	26,713

## VII. Conclusion

The commonly used methods in COVID-19 diagnosis, namely RT-PCR and CT have certain limitations and drawbacks such as long processing times and unacceptably high misdiagnosis rates. These drawbacks are also shared by most of the recent works in the literature based on deep learning due to data scarcity from the COVID-19 cases. Although deep learning-based recognition techniques are dominant in computer vision where they achieved state-of-the-art performance, their performance degrades fast due to data scarcity, which is the reality in this problem at hand. This study aims to address such limitations by proposing a robust and highly accurate COVID-19 recognition approach directly from X-ray images. The proposed approach is based on the CSEN that can be seen as a bridge between deep learning models and representation-based methods. CSEN uses both a dictionary and a set of training samples to learn a direct mapping from the query samples to the sparse support set of representation coefficients. With this unique ability and having the advantage of a compact network, the proposed CSEN-based COVID-19 recognition systems surpass the competing methods and achieve over 98% sensitivity and over 95% specificity. Furthermore, they yield the most computationally efficient scheme in terms of speed and memory.
